# Implantation of a peritoneal dialysis catheter in patients with ESRD using local anesthesia and Remifentanil

**DOI:** 10.1371/journal.pone.0259351

**Published:** 2021-11-04

**Authors:** Elizabeth Jabbour, Carsten Fütterer, Sebastian Zach, Anna-Isabelle Kälsch, Michael Keese, Nuh N. Rahbari, Bernhard K. Krämer, Kay G. Schwenke

**Affiliations:** 1 Department of Surgery, University Medical Center Mannheim, Medical Faculty Mannheim, Heidelberg University, Mannheim, Germany; 2 Department of Anesthesiology, University Medical Center Mannheim, Medical Faculty Mannheim, Heidelberg University, Mannheim, Germany; 3 Department of Vascular Surgery, Universitätsmedizin Mannheim, Medical Faculty Mannheim, Heidelberg University, Mannheim, Germany; 4 Department of Medicine V, University Medical Center Mannheim, Medical Faculty Mannheim, Heidelberg University, Mannheim, Germany; 5 Mannheim Transplantation Center, Universitätsmedizin Mannheim, Medical Faculty Mannheim, Heidelberg University, Mannheim, Germany; 6 European Center for Angioscience, Medical Faculty Mannheim, Heidelberg University, Mannheim, Germany; Ohio State University Wexner Medical Center Department of Surgery, UNITED STATES

## Abstract

**Study objective:**

The main objective of this study is to test the feasibility of the local anesthetic (LA) Mepivacaine 1% and sedation with Remifentanil as the primary anesthetic technique for the insertion of a peritoneal dialysis (PD) catheter, without the need to convert to general anesthesia.

**Methods:**

We analyzed 27 consecutive end-stage renal disease (ESRD) patients who underwent the placement of a peritoneal catheter at our center between March 2015 and January 2019. The procedures were all performed by a general or vascular surgeon, and the postoperative care and follow-up were all conducted by the same peritoneal dialysis team.

**Results:**

All of the 27 subjects successfully underwent the procedure without the need of conversion to general anesthesia. The catheter was deemed prone to usage in all patients and was found to be leak-proof in 100% of the patients.

**Conclusion:**

This study describes a safe and successful approach for insertion of a PD catheter by combined infiltration of the local anesthetic Mepivacaine 1% and sedation with Remifentanil. Hereby, ESRD patients can be treated without general anesthesia, while ensuring functionality of the PD catheter.

## Introduction

The use of peritoneal dialysis has increased by a rate of 14.5% in developing countries and 30.3% in developed countries [1].

Peritoneal dialysis (PD) is the preferred method of dialysis in patients with multiple myeloma, difficulties in fistula generation, coexisting ascites, cardio-renal syndrome, and chronic infections [2]. PD is also beneficial in suitable patients as it facilitates home therapy and increases patient independence [3]. The preserved residual renal function in PD contributes to quality of life and may have additional benefits [4].

However, not every patient is eligible for a PD catheter insertion. The insertion of a PD catheter is contraindicated in patients with any form of abdominal hernia, a large surgical scar or previous complicated intra-abdominal surgeries, recurring diverticulitis, or intellectual disability [5].

The growing number of PD patients with overall increasing comorbidity necessitates the development of fast and safe techniques of PD catheter insertion that avoid use of general anesthesia.

Continuous ambulatory peritoneal dialysis (CAPD) is a well-established form of renal replacement therapy for patients suffering of end stage renal disease (ESRD) [6].

Since its introduction in the late 1960s, the Tenckhoff catheters, and modified versions thereof such as the Oreopoulos-Zellermann catheter, remain the catheters of choice for peritoneal dialysis [7]. The implantation of the catheter can be done using several techniques; the most commonly used method being through a mini laparotomy, i.e. through the opening of the peritoneum [8, 9]. The success of the peritoneal dialysis relies on a leak-proof, adequately positioned catheter, which implies the meticulous placement of the catheter within the peritoneal cavity [10]. Common surgical knowledge is that an opening of the peritoneum necessitates general anesthesia. However, patients with ESRD who do require dialysis are usually multi-morbid, thus they carry a high operative risk [11].

In recent years, the once hypothetical idea of peritoneal opening under local anesthetic infiltration became routine in many operating rooms around the world, saving a multi-morbid ESRD patient from the many risks of intubation [12]. This method is usually done through an ultrasound-guided Transversus abdominis plane (TAP) block, a method which has been thoroughly described and studied, and while feasible holds a high risk of conversion to general anesthesia (GA) [13–17]. Another approach also describes the laparoscopic insertion of the Tenckhoff catheter employing nitrous oxide to create a pneumoperitoneum under local anesthetic infiltration [18]. In the last decade, many non-surgical methods of PD catheter insertion have emerged. However, the blind Seldinger technique has faced numerous controversies, especially due to the risk of bowel perforation. In recent years, this method has been developed to include a blunt hollow introducer accompanied by blunt dilators that allowed a guidewire insertion of the Catheter [19].

Mepivacaine is used as the local anesthetic (LA) of choice for its unique features, its amide structure (therefore it is not detoxified by circulating plasma esterases) and its quick metabolism through the liver. Clinically, Mepivacaine shows a short onset time, one very similar to Lidocaine, intermediate duration and low toxicity. Mepivacaine can be therefore considered as a safe choice for local anesthesia, particularly in high risk patients [20]. Remifentanil is a known selective μ-agonist opioid of the phenylpiperidine group, with an approximately 10-minute excretion half-life [21].

## Materials and methods

### Ethic statement

An approval of the ethics committee (Medizinische Ethik-Kommission II der Ruprecht-Karls-Universität Heidelberg, Medizinische Fakultät Mannheim, Universitätsklinikum Mannheim, 831R-19) was obtained for the current study. Written consent was signed by all patients.

### Study design

This is a prospective single center cohort feasibility study. Primary end point is successful PD after 14 days. Secondary end points are conversion to general anesthesia, complications, leakage and procedure time.

### Patients and data collection

We screened 31 consecutive patients who needed to receive peritoneal catheter insertion in the university hospital of Mannheim in Germany between March 1, 2015 and January 31, 2019.

Our exclusion criteria included previous major abdominal surgeries, patients below the age of 18, patients with known allergies to Mepivacaine or Remifentanil, and patients who did not consent to undergoing the procedure under local anesthetic and sedation.

All patients had a clear clinical diagnosis of underlying ESRD and required renal replacement therapy prior to a PD catheter insertion. The clinical diagnosis and therefore the eligibility for CAPD and the indication for catheter placement were determined by various nephrologists at the university hospital in Mannheim. The patients were therefore referred to the department of surgery to undergo the procedure.

All patients selected for this procedure were examined by a surgeon and an anesthesiologist before the indication for operation. We included 27 patients in this study. 4 patients were not included due to meeting exclusion criteria (one patient did not consent to surgery without general anesthesia, one patient was a minor at age 16, and two patients had peritonitis due to bowel perforation in the past medical history).

Demographic information and information regarding the surgical procedure were recorded.

An anesthesiologist performed a standardized sedation to accompany the LA infiltration. The sedation method used throughout the operation was a Remifentanil perfusor at a rate of 0.1μg /kg/min. The dose of Remifentanil was not increased beyond the initial recommended dose of 0.1 μg /kg/min. The unplanned conversion to general anesthesia was considered necessary at the discretion of the treating anesthesiologist. The criteria for transition to general anesthesia included uncontrollable patient movement, patients complaint of pain, patients wish to convert, coma, apnea or loss of airway protection due to Remifentanil. Intra-operative sedation and local anesthetic infiltration efficacy was determined by the individual response and overall patient tolerance. Mepivacaine was adapted accordingly without exceeding the maximum recommended weight adapted dose. The local anesthetic infiltration was performed by or under the direct supervision of an experienced consultant for either vascular or general surgery. The patient was continuously monitored by a senior anesthesiologist.

### The operative procedure

**Fig 1:**
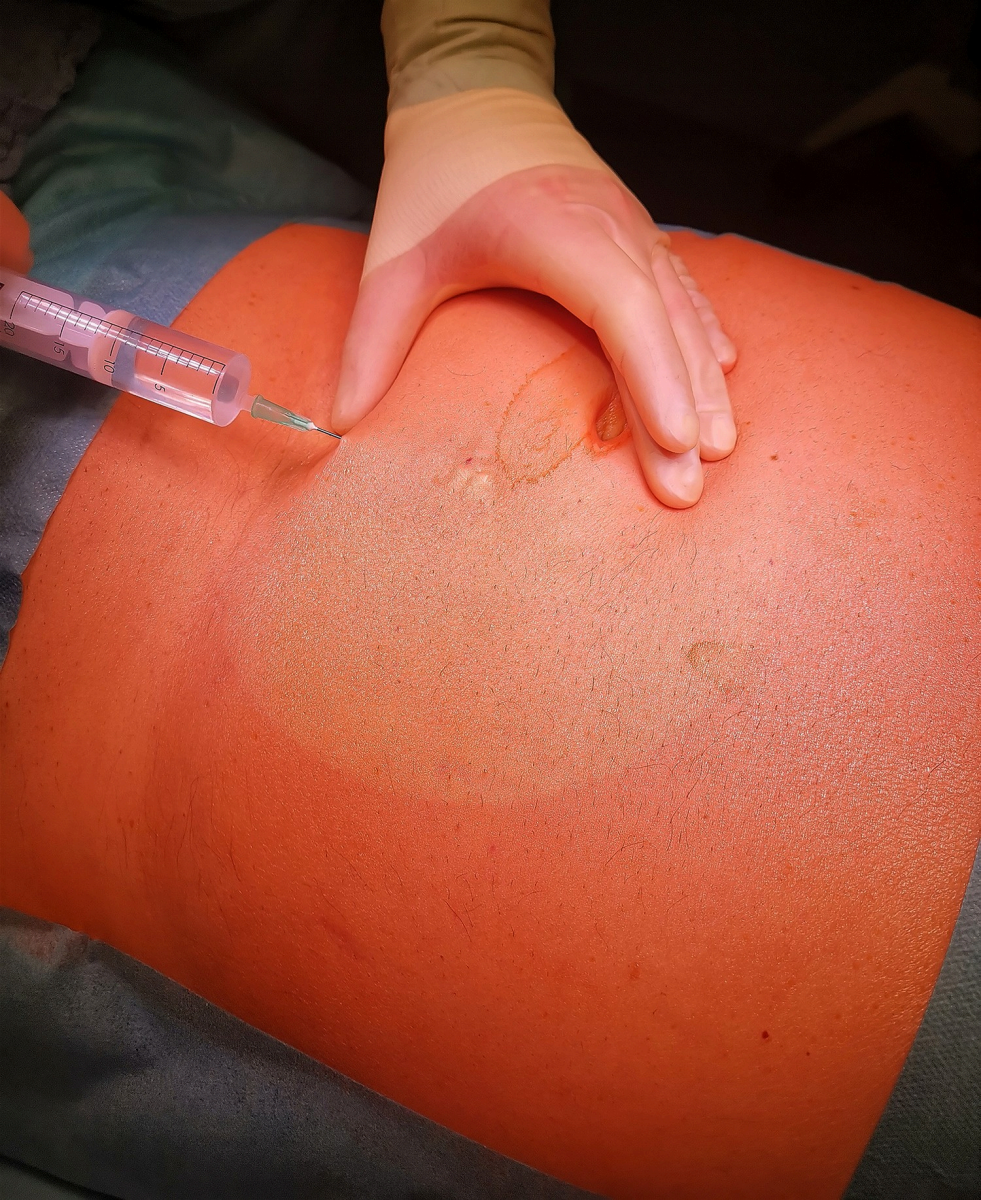
BMI-adapted (depth of tissue from skin to fascia) infiltration of the surgical site with 10–15 ml Mepivacaine 1%.

All procedures were performed by a general or vascular surgeon. After weight- and size adapted (depth of tissue from skin to fascia) infiltration of the surgical site with 10–15 ml Mepivacaine 1% (Fig 1), a 6–8 cm para-median skin incision is carried out to expose the anterior rectus sheath. 10–15 ml Mepivacaine infiltration into the Rectus sheath is followed by longitudinal incision of the anterior Rectus sheath. After blunt lateralization of the Rectus muscle, a longitudinal incision on the posterior wall of the rectus-sheath is performed.

**Fig 2:**
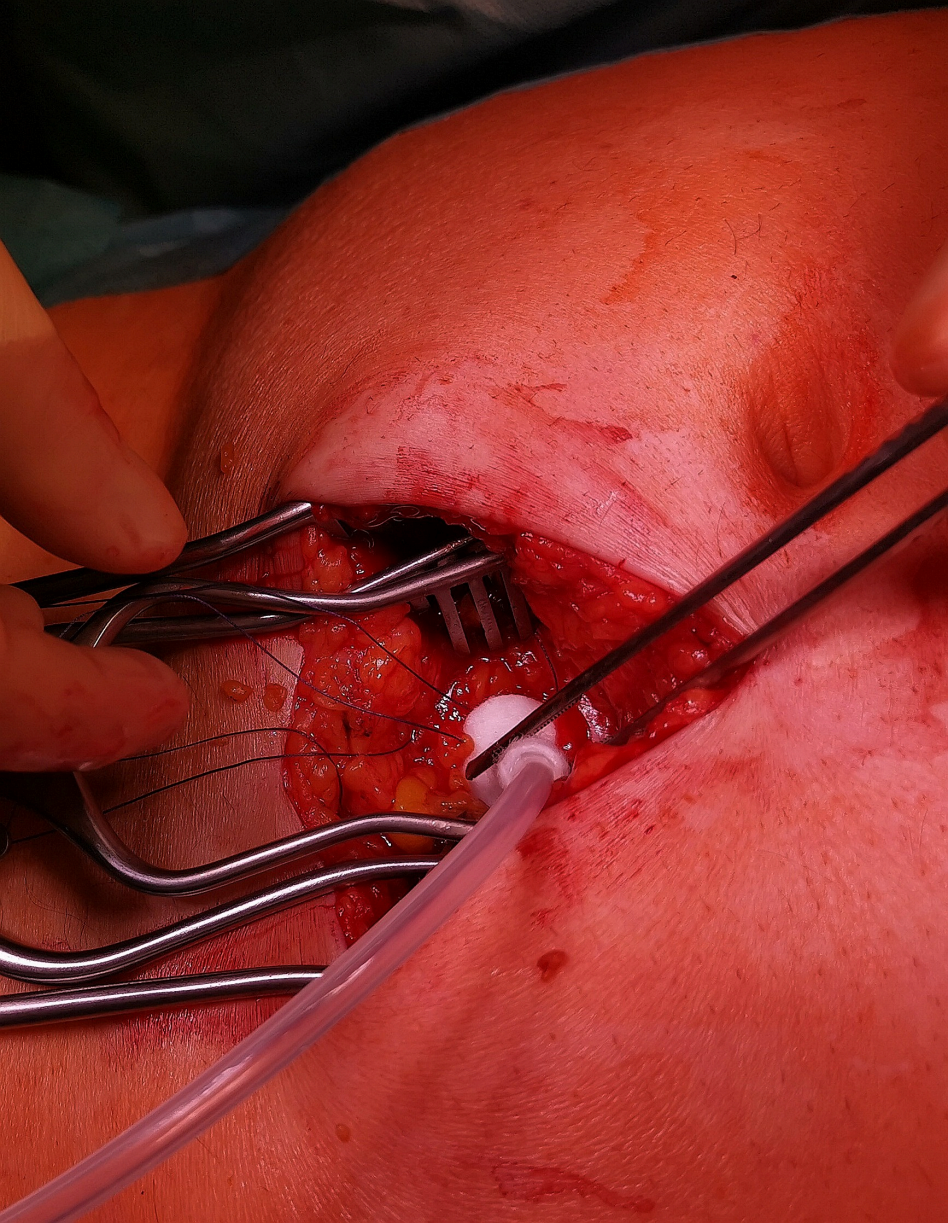
The catheter is firmly anchored at the silicone bead by closing the peritoneal purse string suture.

**Fig 3:**
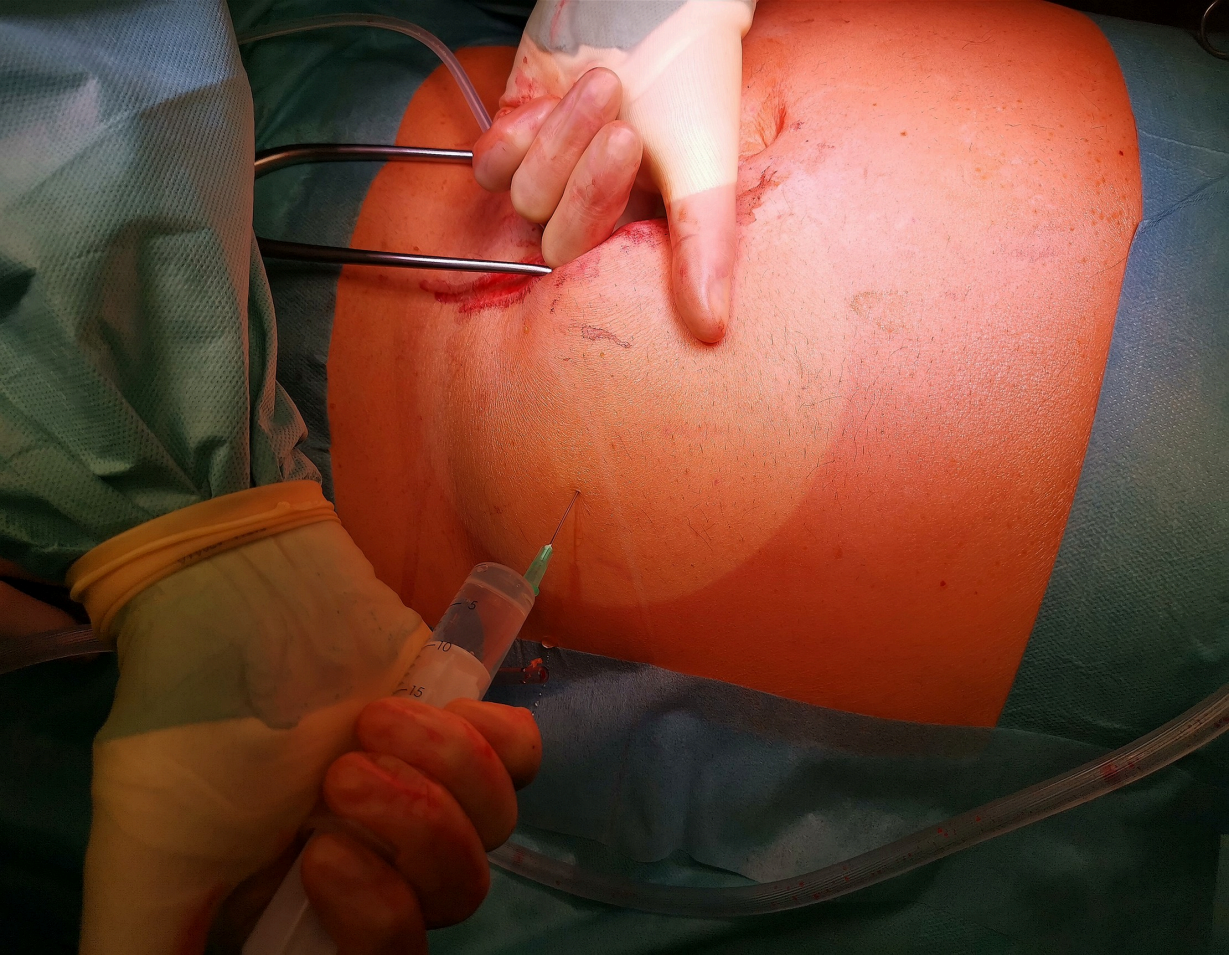
After infiltration at the region of the planned catheter exit point, a depot of 2–5 ml Mepivacaine 1% is applied.

Before opening the peritoneum right above the arcuate line, another 5–10 ml of Mepivacaine are injected to place a purse-string suture (Vicryl 3.0) on the peritoneum. The PD catheter is introduced via a small incision within the purse-string suture and placed in the Douglas space using dressing forceps. The catheter is firmly anchored at the silicone bead by closing the peritoneal purse string suture (Fig 2). The posterior rectus sheath is closed by running suture (PDS 2.0) on top of the Dacron cuff. After infiltration at the region of the planned catheter exit point, a depot of 2–5 ml Mepivacaine 1% is applied (Fig 3). The catheter is then placed posterior to the Rectus muscle and through the planned exit point. Thereafter, the anterior Rectus sheath (PDS 0) is closed. The function and possible leakage of the catheter are then tested with the inflow and outflow of 250 ml saline. After closure of the subcutaneous layer and the Cutis, the catheter is prone to usage immediately.

### Statistical analysis

Statistical analysis was performed using statistical software (SPSS for windows, Version 20, SPSS science, IBM). The characteristics of patients with ESRD were summarized using proportions and medians. Categorical values are presented in tables as number of cases with the percentage of cases given in parenthesis. Continuous variables are described as mean ± standard deviation (SD). Data was compared between 27 consecutive ESRD patients with different demographics.

## Results

In the study period from March 2015 to January 2019, 27 consecutive patients underwent open surgical PD catheter insertion. All patients were indicated for peritoneal dialysis. In all patients, the catheters could be placed under the local infiltration of pain- and response-adapted local anesthetic and a sedation regimen with the use of a Remifentanil perfusor at a rate of 0.1μg/kg/min. Between 30 and 40 ml of Mepivacaine 1% were used, depending on body weight.

Demographic information of all patients is provided in Table 1.

**Table 1:** Demographic data.

Demographic Category	Patients (N = 27)
Sex	
Male	20/27 (74.08%)
Female	7/27 (25.92%)
Age, mean ± SD, years	69.96 ± 14.82 (range 29–90)
BMI, mean ± SD, kg/m^2^	27.27 ± 5.74 (range 18.6–44.8)
ASA score, mean ± SD	3.63 ± 0.76
ASA 3	10 (37.1%)
ASA 4	17 (62.9%)

The mean age of our patients was 69.96 (SD 14.82, range 29–90). The PD catheter was inserted in 20 males and 7 females. The average BMI of our patients was 27.27 kg/m^2^ (SD 5.74, range 18.6–44.8). The Average ASA (American Society of Anesthesiologists) score, defined as the score that assesses the physical status of patients before surgery [22], was at 3.63 (SD 0.77, Range 3–4) [Table 1].

Information regarding the surgical procedure itself (success, time, conversion to general anesthesia) and postoperative factors (wound infection, stability of the PD catheter including leakage rate) are given in Table 2.

**Table 2:** Intra- and postoperative outcomes (at 30 days).

Factors	Patients (N = 27)
Leakage	0/27 (0%)
Mortality or complication	0/27 (0%)
Surgical wound infection, (CDC A1)	1/27 (3.7%)
Conversion to GA	0/27 (0%)
Duration of the procedure, mean ± SD, min	35.59 ± 7.403 (range 21–56)

The combined regimen with the local infiltration with Mepivacaine 1%, as well as sedation, provided successful surgical anesthesia in all 27 patients (100% of the cases) with no need to convert to general anesthesia. No severe discomfort, complaint or dissatisfaction with the procedure was noted by any of the subjects questioned during and after surgery.

No adverse reactions to Mepivacaine 1%, such as intravascular administration or anaphylactic shock, were recorded [23]. The average procedure duration (operation and local anesthesia) in minutes was 35.59 (SD 7.40, range 21–56). All patients were successfully able to start the peritoneal dialysis within 14 days after the procedure.

## Discussion

Our study shows the feasibility of inserting a PD catheter with a combined infiltration of the local anesthetic Mepivacaine 1% and sedation with Remifentanil as all 27 subjects included in this study underwent the procedure without the need to convert to general anesthesia. Previous studies have shown a successful insertion or removal of the PD catheter using an Ultrasound-guided TAP block performed mainly by anesthesiologists, where in 24 patients, 21 underwent the procedure without conversion to general anesthesia, with a success rate of 87.5% [13].

The results of our study provide a new approach in the use of local anesthetic infiltration without any time-consuming preoperative ultrasound guidance as previously described in the TAP block.

Superficial incisional surgical wound infection (A1) was observed in 1 of the 27 subjects (3.7%). However, the rate of wound infection after PD catheter insertion using local anesthetic infiltration as a main blockade, a procedure considered to be a clean surgery without a break in sterile technique, was still lower than the 8% measured in the general population who underwent any other clean surgery in which neither the alimentary, respiratory, or genitourinary tracts have been penetrated [24].

The study further shows a 0% catheter leakage rate, which proves the efficacy of the operative technicality. [Table 2].

One of the main strengths of our technique is also a relevant reduction in procedure time, with an average of 35.59 ((SD 7.403, range 21–56), [Table 2] in comparison to a much lengthier procedure when the ultrasound-guided TAP block has been performed, ranging from anywhere between 51 to 259 minutes [13, 15].

Although the percutaneous insertion of the catheter was deemed successful in providing peritoneal access, the method is still criticized because of high incidence of leakage, mechanical complications and its potential risks of intraabdominal injury in being a ’’blind’’ method that does not allow a proper visualization of the peritoneum [25].

Furthermore, the advantages of a surgical insertion using this minimally invasive method in comparison to the percutaneous placement method are: better tissue retraction, lesser mechanical complications and therefore the exclusion of bowel injury [26]. Moreover, the percutaneous insertion of peritoneal dialysis catheters is still contraindicated in morbidly obese patients with a BMI >35 [27]. In our study, the catheter was successfully inserted into a patient with a BMI of 44.8 kg/m2.

The main limitation of this study is the inability to adequately assess if the local LA infiltration was sufficient by itself, or if only the addition of sedatives administered by the anesthesiologists was responsible for the high success rate observed in pain management, thus omitting the need to convert to general anesthesia.

## Conclusion

The use of Remifentanil and Mepivacaine in our suggested setting furnishes a safe and fast approach to provide sufficient anesthesia to successfully insert a peritoneal dialysis catheter in ESRD patients without need for general anesthesia.
